# Correction

**DOI:** 10.1080/15384047.2024.2350871

**Published:** 2024-05-05

**Article title**: ERG oncogene modulates prostaglandin signaling in prostate cancer cells

**Authors**: Ahmed A. Mohamed, Shyh-Han Tan, Chen Sun, Syed Shaheduzzaman, Ying Hu, Gyorgy Petrovics, Yongmei Chen, Isabell A. Sesterhenn, Hua Li, Taduru Sreenath, David G. McLeod, Albert Dobi, Shiv Srivastava

**Journal**: Cancer Biology & Therapy

**DOI**: https://doi.org/10.4161/cbt.11.4.14180

The author brought to our attention that Figure 2 has no background, suggesting that images of the same band has been re-used in the figure. They provided Figure 2 with the pixelate background to clarify this concern. Further, the negative result of KLK3 IgG experiment is provided in the correct orientation.

**Figure 2A.**
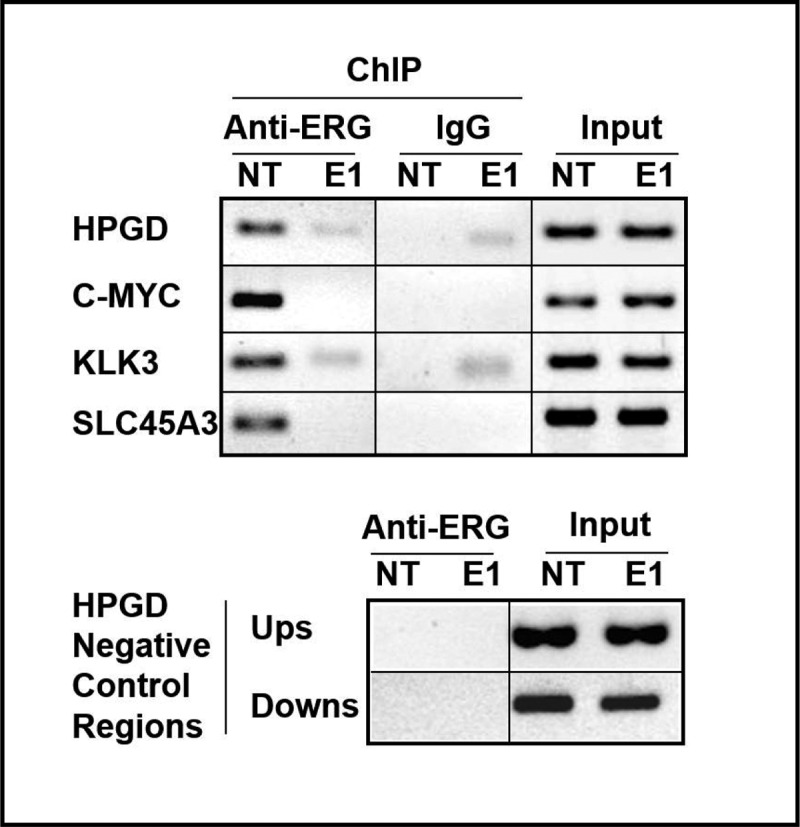

They also informed that Figure 4B, the loading control image was an inadvertent duplicate of Figure 4A. They provided Figure 4B with the correct loading control image.

**Figure 4B.**
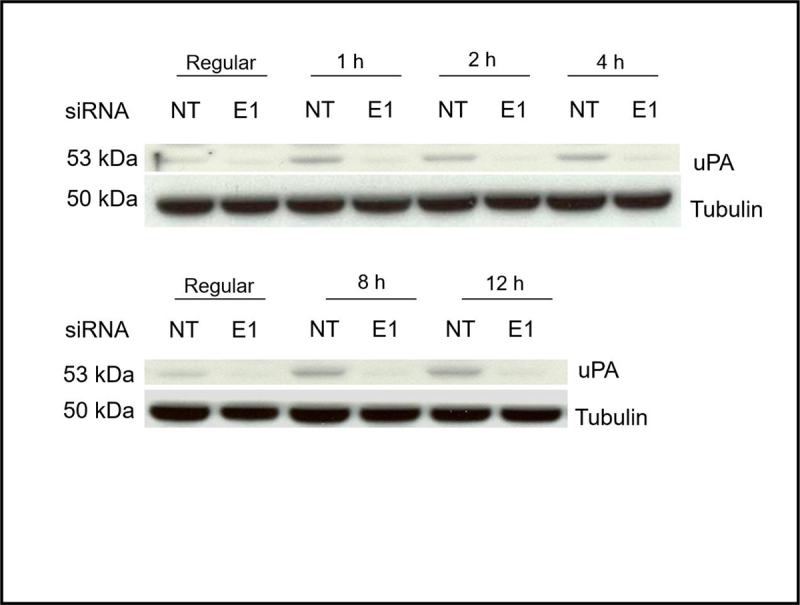

These corrections do not change the results and interpretation of the article.

Correct versions of figures are as below.

